# A model for rapid PM_2.5_ exposure estimates in wildfire conditions using routinely available data: rapidfire v0.1.3

**DOI:** 10.5194/gmd-17-381-2024

**Published:** 2024-01-16

**Authors:** Sean Raffuse, Susan O’Neill, Rebecca Schmidt

**Affiliations:** 1 Air Quality Research Center, University of California, Davis, Davis, CA, United States; 2 Pacific Northwest Research Station, USDA Forest Service, Seattle, WA, United States; 3 Department of Public Health Sciences, MIND Institute, University of California Davis School of Medicine, Davis, CA, United States

## Abstract

Urban smoke exposure events from large wildfires have become increasingly common in California and throughout the western United States. The ability to study the impacts of high smoke aerosol exposures from these events on the public is limited by the availability of high-quality, spatially resolved estimates of aerosol concentrations. Methods for assigning aerosol exposure often employ multiple data sets that are time-consuming to create and difficult to reproduce. As these events have gone from occasional to nearly annual in frequency, the need for rapid smoke exposure assessments has increased. The rapidfire (relatively accurate particulate information derived from inputs retrieved easily) R package (version 0.1.3) provides a suite of tools for developing exposure assignments using data sets that are routinely generated and publicly available within a month of the event. Specifically, rapidfire harvests official air quality monitoring, satellite observations, meteorological modeling, operational predictive smoke modeling, and low-cost sensor networks. A machine learning approach, random forest (RF) regression, is used to fuse the different data sets. Using rapidfire, we produced estimates of ground-level 24 h average particulate matter for several large wildfire smoke events in California from 2017–2021. These estimates show excellent agreement with independent measures from filter-based networks.

## 1 Introduction

Changes in climate in the western United States, and elsewhere, are driving larger, more intense fires with greater smoke impacts on larger populations (Burke et al., 2021), and these trends are projected to continue (Hurteau et al., 2014). The wildfire seasons of 2020 and 2021 produced some of the highest concentrations of particulate matter, less than 2.5 μm in diameter (PM_2.5_), ever observed in monitoring stations around California, some for several days or weeks. Despite reductions in ambient PM_2.5_ driven by air pollution regulations, areas of the western United States are seeing increasing concentrations due to wildfire smoke impacts (McClure and Jaffe, 2018).

There are widespread concerns about potential health consequences of wildfire exposures on vulnerable populations as the smoke increasingly reaches populated areas. From 2008–2012, it was estimated that over 10 million individuals in the United States experienced unhealthy air quality levels (average daily fire PM_2.5_ > 35 μg m^−3^) associated with exposure to wildfire for more than 10 d (Rappold et al., 2017). This number is expected to have risen several-fold in the decade since, given the increase in wildfire events across the continent (Childs et al., 2022). Additionally, long-range transport of wildfire PM_2.5_ has been associated with adverse health effects in susceptible populations thousands of miles away (Le et al., 2014; Kollanus et al., 2016).

Wildfire smoke is associated with premature deaths (Chen et al., 2021a; Johnston et al., 2012) and significant cardiovascular (Chen et al., 2021b) and respiratory morbidity (Reid et al., 2016), including asthma exacerbations. Certain subpopulations are more susceptible to the health impacts of air pollution and wildfire smoke, including the elderly, pregnant women, and those with underlying health conditions such as asthma (Chen et al., 2021b). Few studies have examined long-term health outcomes in relation to chronic exposures to high concentrations of wildfire smoke. Prenatal wildfire smoke exposure has been linked to adverse birth outcomes, including preterm birth (Heft-Neal et al., 2022) and lower birth weight (Abdo et al., 2019; Holstius et al., 2012), especially with exposure in the second or third trimester. In contrast to studies of ambient air pollution, associations between wildfire smoke and adverse birth outcomes did not differ by race, ethnicity, or income but differed by baseline smoke exposure. Many epidemiological studies have linked early-life air pollution exposure to increased autism spectrum disorder risk (Volk et al., 2013; Dutheil et al., 2021; Volk et al., 2011) and to cognitive functioning impairments (Clifford et al., 2016; Loftus et al., 2019; Chiu et al., 2016; Loftus et al., 2020).

Evidence suggests that wildfire PM_2.5_ could induce higher toxicity than other ambient air PM_2.5_ (Kim et al., 2018; Wegesser et al., 2010; Franzi et al., 2011; Wegesser et al., 2009) and is associated with about 10 times higher increase in hospital admissions for respiratory health than PM_2.5_ from other sources (Aguilera et al., 2021a), including in young children (Aguilera et al., 2021b). With climate predictions for increased occurrence and severity of wildfires, there is a growing need to understand which populations are at highest risk and PM_2.5_ concentrations of concern to inform adverse-health mitigation strategies. Yet, many gaps remain in our understanding of the linkages between wildfire smoke and human health (Black et al., 2017). A critical challenge is in characterizing personal or population exposures during high-intensity events. There are many methods for estimating exposure to ambient pollution, including spatial interpolation of measured values, chemical transport modeling, remote sensing, land-use regression modeling, data fusion and machine learning, and combinations of all of these approaches (e.g., Reid et al., 2015; Zhang et al., 2020; Al-Hamdan et al., 2014; Cleland et al., 2020; Hoek et al., 2008). The rapidly changing conditions during wildfire smoke events can confound otherwise high-performing approaches (O’Neill et al., 2021). There are several barriers to the adoption of existing methods for exposure assignment. These can include data availability for the study location, data latency, and high-performance computing requirements. The combination of increasing frequency of smoke events and the proliferation of smoke exposure human health studies drives a need for exposure modeling that is quick and inexpensive.

There has been a rapid proliferation of low-cost sensors for air quality within the past decade. While these sensors do not measure PM_2.5_ with the same fidelity as the regulatory monitoring conducted by federal and local air quality agencies, they represent a new resource for PM_2.5_ assessment with relatively dense spatial coverage. Many low-cost PM_2.5_ sensors operate with similar principles, using a laser to count particles that scatter light in the optical range, with sensitivities peaking for aerosols with median scattering diameter of < 0.3 μm (Ouimette et al., 2022). Recent studies have shown the value of incorporating low-cost sensor networks into PM_2.5_ exposure modeling (Bi et al., 2020).

Past work has shown that a data fusion approach that combines ground-based air quality monitors, transport modeling that incorporates wildfire emissions, satellite observations, and meteorological variables can be effective in predicting PM_2.5_ exposure during large wildfire events (Zou et al., 2019; O’Neill et al., 2021) and prescribed fires (Huang et al., 2021).

We developed methods and a suite of tools for rapidly predicting PM_2.5_ exposure, particularly during wildfire smoke events, using readily available data with low latency (less than 1 month). The tools are contained within a package written in the R programming language called rapidfire (relatively accurate particulate information derived from inputs retrieved easily). rapidfire adapts and builds upon the methods of Zou et al. (2019) and O’Neill et al. (2021), replacing retrospective chemical transport modeling and other data sets developed for research with smoke forecast modeling and “off-the-shelf” data sets that are routinely available and easily acquired. A major addition is the incorporation of low-cost sensor data. This paper describes the data sets and algorithms used in the rapidfire package and presents an example case study during five recent extreme wildfire seasons in California.

## 2 Methods

**Figure 1.**
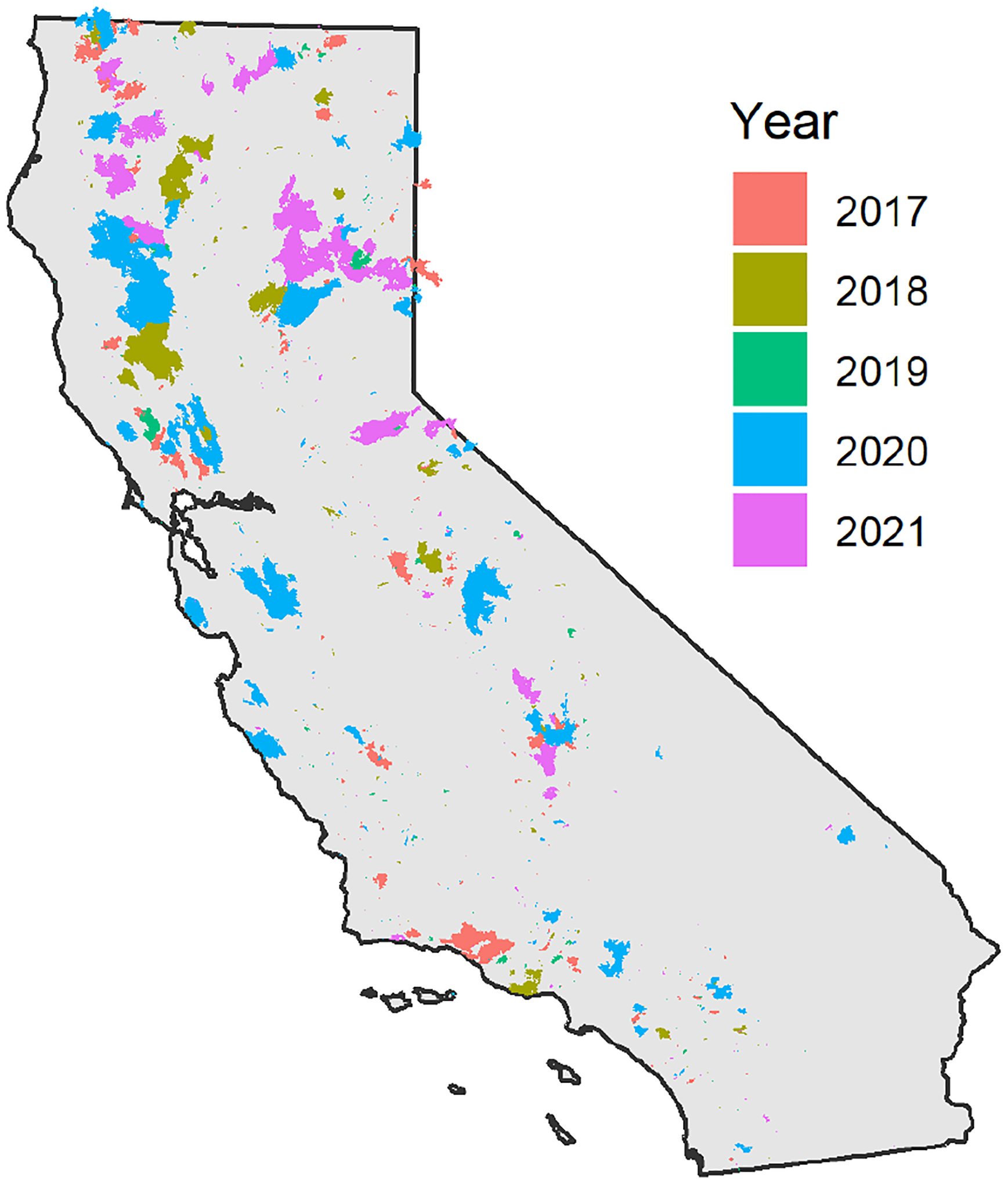
Locations of burned areas in California, 2017–2021.

**Figure 2.**
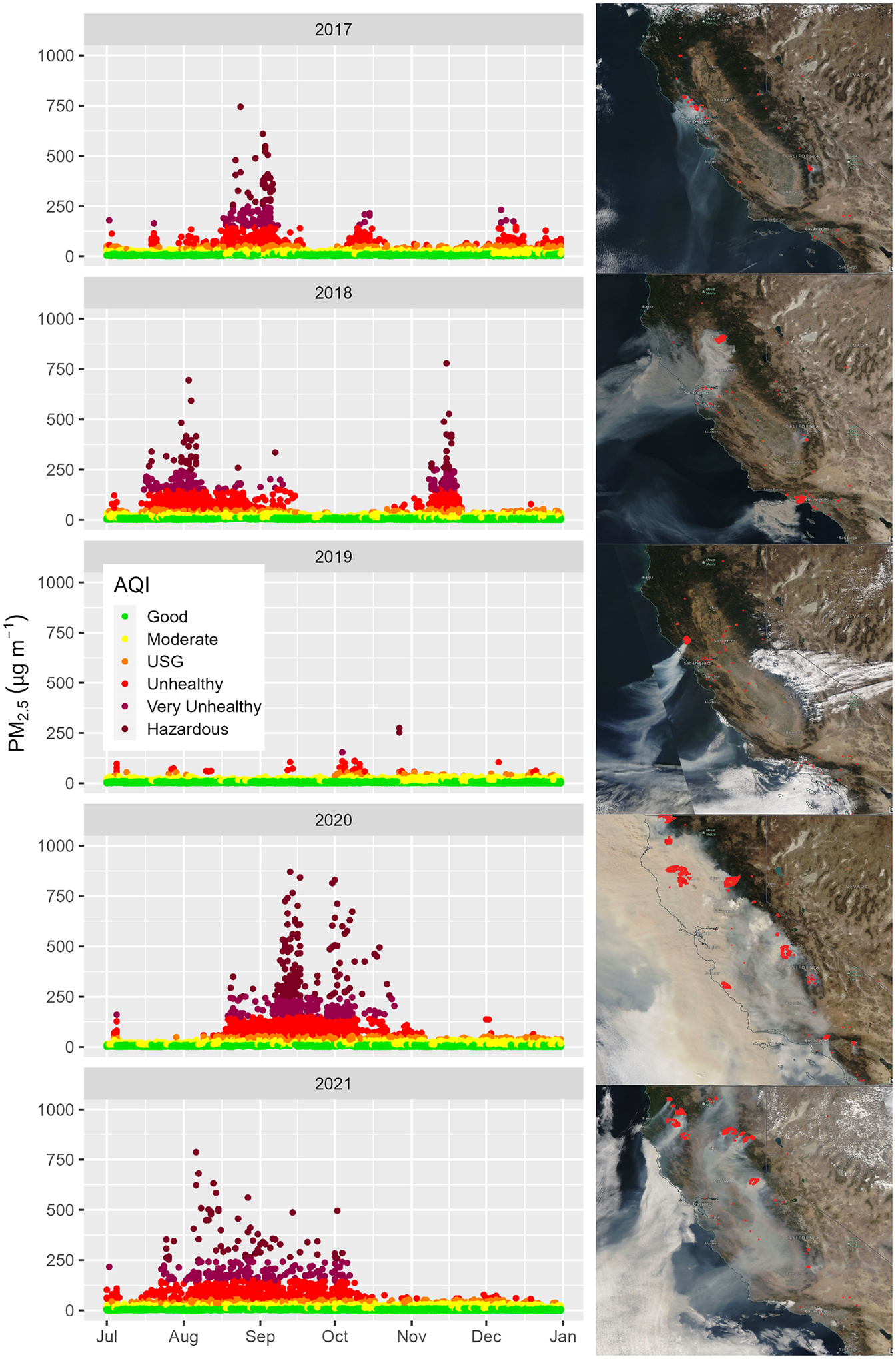
Temporal and area views of smoke impacts across California. Panels on the left show 24 h PM_2.5_ concentrations from permanent and temporary monitors in California for July–November for 2017–2021. Data are color-coded by air quality index. Panels on the right show visible satellite imagery of smoke and satellite fire hotspot detections across California from NASA Worldview for 13 October 2017 during the wine country wildfires, 9 November 2018 during the Camp and Woolsey wildfires, 27 October 2019 during the Kincade wildfire, 9 September 2020 after widespread lightning ignition of wildfires in northern and central California, and 19 August 2021 when many wildfires were burning in northern California and the Sierras.

In this study, data sets and algorithms are applied to time periods of large California wildfires from 2017–2021. Table 1 summarizes some of the major California wildfires and the area burned for the year. Figure 1 shows the wildfire locations, as detailed by the California Department of Forestry and Fire Protection’s Fire and Resource Assessment Program (FRAP). Extreme fire weather conditions fueled the October 2017 wine country wildfires (~ 81 ha) in the Napa and Sonoma counties of central coastal California (Mass and Ovens, 2019), and over 7 million people were impacted by unhealthy levels of smoke (O’Neill et al., 2021). The 2018 wildfire season began in July with wildfires such as the Carr, Ferguson, and Mendocino Complex (Mueller et al., 2020) and extended through November with the Camp and Woolsey wildfires. In comparison, 2019 was a relatively low-activity fire year, but the Kincade wildfire (~ 31 ha) again impacted the wine country in October–November. The 2020 wildfire season was relatively quiet until the middle of August when widespread lightning ignited many wildfires across central and northern California, including the coastal range south of San Francisco. In 2021 about two-thirds of the acres were burned as in 2020, but over a longer duration, starting about a month earlier in July. These different patterns and the level of smoke impacts are seen in Fig. 2, which shows 24 h average PM_2.5_ concentrations from permanent and temporary monitors across the state of California and satellite imagery of the smoke and satellite hotspot detections.

### 2.1 Input data sets

Input data for rapidfire consist of ground-based monitors from three sources, aerosol optical depth from satellite instruments, and modeled meteorological and air quality data. Table 2 summarizes these data sources and the rapidfire functions used to access them and/or the location where the data can be obtained.

#### 2.1.1 Permanent and temporary air quality monitoring data

Hourly PM_2.5_ observations are available from monitoring stations across the United States via the AirNow program, which is a partnership of the United States Environmental Protection Agency (EPA); National Oceanic and Atmospheric Administration; National Park Service; NASA; Centers for Disease Control and Prevention; and tribal, state, and local air quality agencies (https://www.airnow.gov/, last access: 10 January 2024). Within California, about 117–141 monitors were operating during the study period. These permanent monitors are a mixture of federal reference method or federal equivalent method instruments, instruments of sufficient quality such that the data are used by EPA to determine attainment and non-attainment of the National Ambient Air Quality Standards (NAAQS).

**Figure 3.**
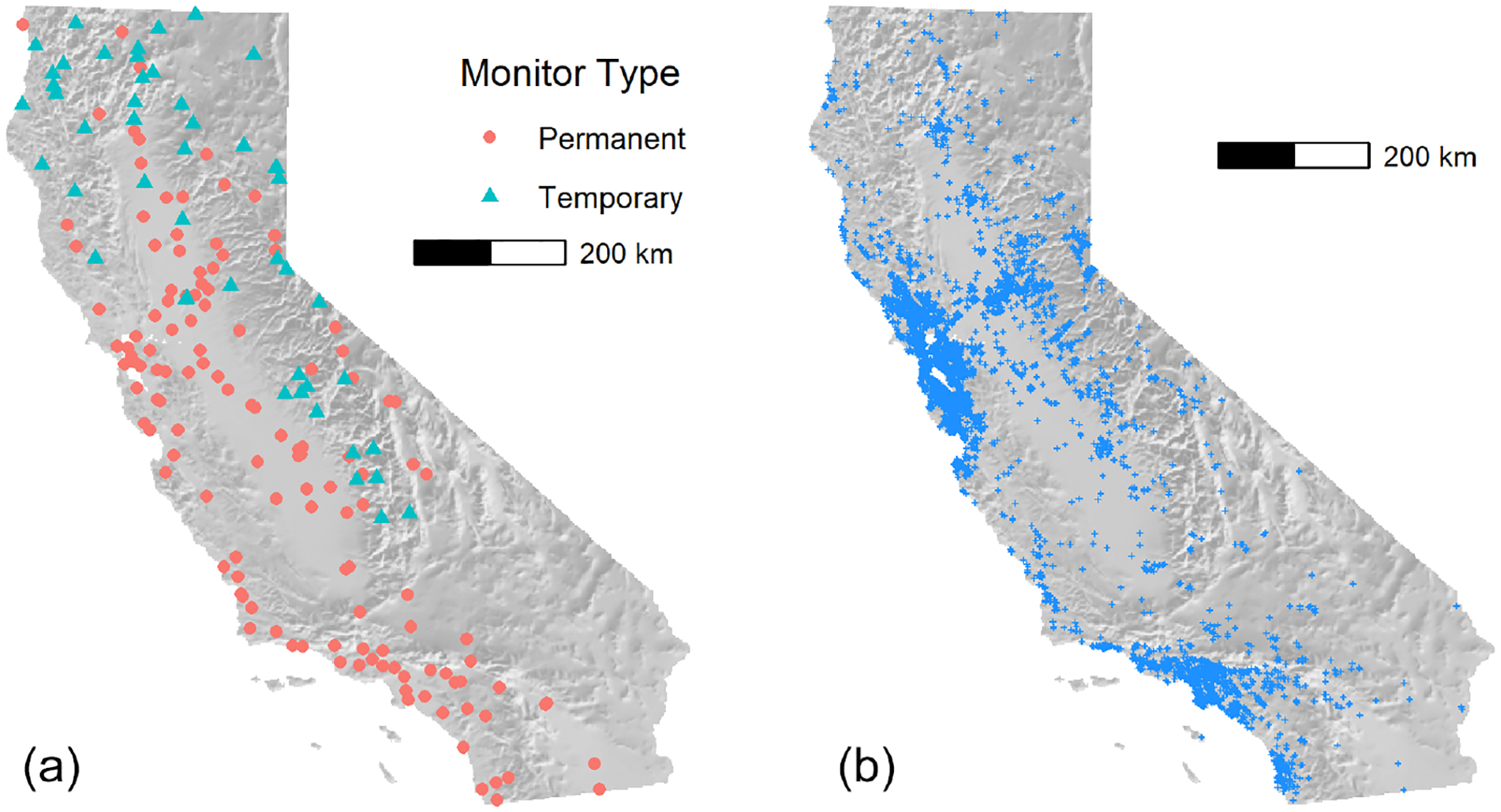
Map of permanent and temporary California monitor locations **(a)** and PurpleAir outdoor sensor locations **(b)**, 1 September 2021.

During wildfires, temporary monitors are also deployed by the Interagency Wildland Fire Air Quality Response Program (IWFAQRP; Congress.gov, 2019) and the California Air Resources Board (CARB). These monitors are environmental beta attenuation monitors (E-BAMs; Met One Instruments, Inc.). As discussed in O’Neill et al. (2021), laboratory (Trent, 2006) and field (Schweizer et al., 2016) studies, evaluating E-BAM performance with federal reference method monitors (BGI Inc., PQ-200, and Met One Instruments BAM) found correlations greater than 0.9 with a tendency of the E-BAMs to overestimate PM_2.5_, especially when relative humidity was greater than 40 % (Schweizer et al., 2016). Though not as accurate as the AirNow monitors, they are deployed in regions where smoke impacts are significant and permanent monitoring is sparse or absent. The locations of permanent and temporary monitors as of 1 September 2021 are shown in Fig. 3 (left). The permanent monitors are concentrated in the coastal and valley regions where larger populations of people are located, while temporary monitors are focused in areas of complex terrain where most wildfires and smaller communities without air quality monitoring data are located.

Hourly PM_2.5_ concentrations from both the permanent and temporary monitors were acquired using the rapidfire::get_airnow_daterange and rapidfire::get_airsis_daterange functions. These wrap the monitor_subset function from the Mazama Science PWFSLSmoke R package (Mazama Science, 2024). rapidfire::recast_monitors was then used to calculate daily 24 h averages from the hourly data. At least 16 h is required to produce an average. The daily average data from both the permanent and temporary monitors were combined into a single data set. Of this monitor data set, 30 % was withheld for development and evaluation of the rapidfire model results. The remaining 70 % was used to develop model variograms using rapidfire::create_airnow_variograms. These PM_2.5_ observations were then log-transformed and interpolated to estimate concentrations at locations away from the monitors using ordinary kriging (Wackernagel, 1995), providing a spatially complete data set for use in the rapidfire data fusion.

#### 2.1.2 Low-cost sensors

There has been a proliferation of low-cost sensors that estimate PM_2.5_ deployed by the public across the world in the last decade. We used data from the PurpleAir network, which had grown to over 6500 outdoor sensors in California as of the end of 2021. Figure 3 (right) shows the locations of PurpleAir sensors reporting data on 1 September 2021. Coverage in populated areas is extensive.

While PurpleAir estimates of PM_2.5_ concentration have been shown to be biased and are dependent on humidity and aerosol type (Barkjohn et al., 2021), they still correlate with PM_2.5_ observed at FEM monitors and provide invaluable spatial and temporal information that is not available with the relatively sparse network of monitors. Because these sensors are not quality controlled or validated, and their siting may be suspect, care must be taken when using them in modeling.

For time periods since February 2021, rapidfire acquires PurpleAir archive data using the OpenAQ application programming interface (API). OpenAQ is a non-profit data platform that aggregates air quality data from around the world (OpenAQ, 2023). rapidfire::openaq_find_sites is first run to find all sensors within a specified geographic boundary. Then, rapidfire::openaq_get_averages can be used to download data for those sensors over the specified time period. At the time of publication, PurpleAir data from prior to February 2021 were not available via OpenAQ. For earlier time periods, rapidfire queries data directly from the PurpleAir API. rapidfire::pa_find_sensors is used for finding all available outdoor PurpleAir sensors within a geographic bounding box. Then, rapidfire::pa_sensor_history can be run to acquire hourly PM_2.5_ concentration estimates from each sensor. Note that access to historical data via the PurpleAir API now requires an API key, and there is a cost for requesting larger amounts of data. There is no cost to access the data via OpenAQ.

We employ a spatial test to remove sensors that are significantly different from their neighbors. rapidfire::purpleair_clean_spatial_outliers removes any sensors that are more that 2 standard deviations away from the median of all sites within 10 km. PurpleAir estimates used in data fusion were log-transformed and then interpolated using ordinary kriging. While it is common to apply a correction to PurpleAir data to better correlate with PM_2.5_ from standard monitors, we elected not to do so. The data fusion model described below incorporates relative humidity and other meteorological parameters and is, in essence, applying a correction specific to the region and time period of the modeling domain.

#### 2.1.3 Satellite aerosol optical depth

Satellite aerosol optical depth (AOD) is a measure of the total columnar aerosol light extinction from the satellite sensor to the ground. AOD is indirectly related to PM_2.5_, with the relationship depending on aerosol type, humidity, and aerosol vertical profile (Li et al., 2015). We used AOD from the Multi-Angle Implementation of Atmospheric Correction (MAIAC) project (Lyapustin et al., 2011). MAIAC is an advanced algorithm that uses time series analysis and additional processing to improve aerosol retrievals; atmospheric correction; and, importantly, cloud detection from the Moderate Resolution Imaging Spectroradiometer (MODIS) instruments on board NASA’s Terra and Aqua satellites. Past work has shown that thick smoke is often mistaken for clouds in the standard MODIS algorithms (van Donkelaar et al., 2011), which hampers their use in wildfire conditions. The MAIAC algorithm reduces those errors.

**Figure 4.**
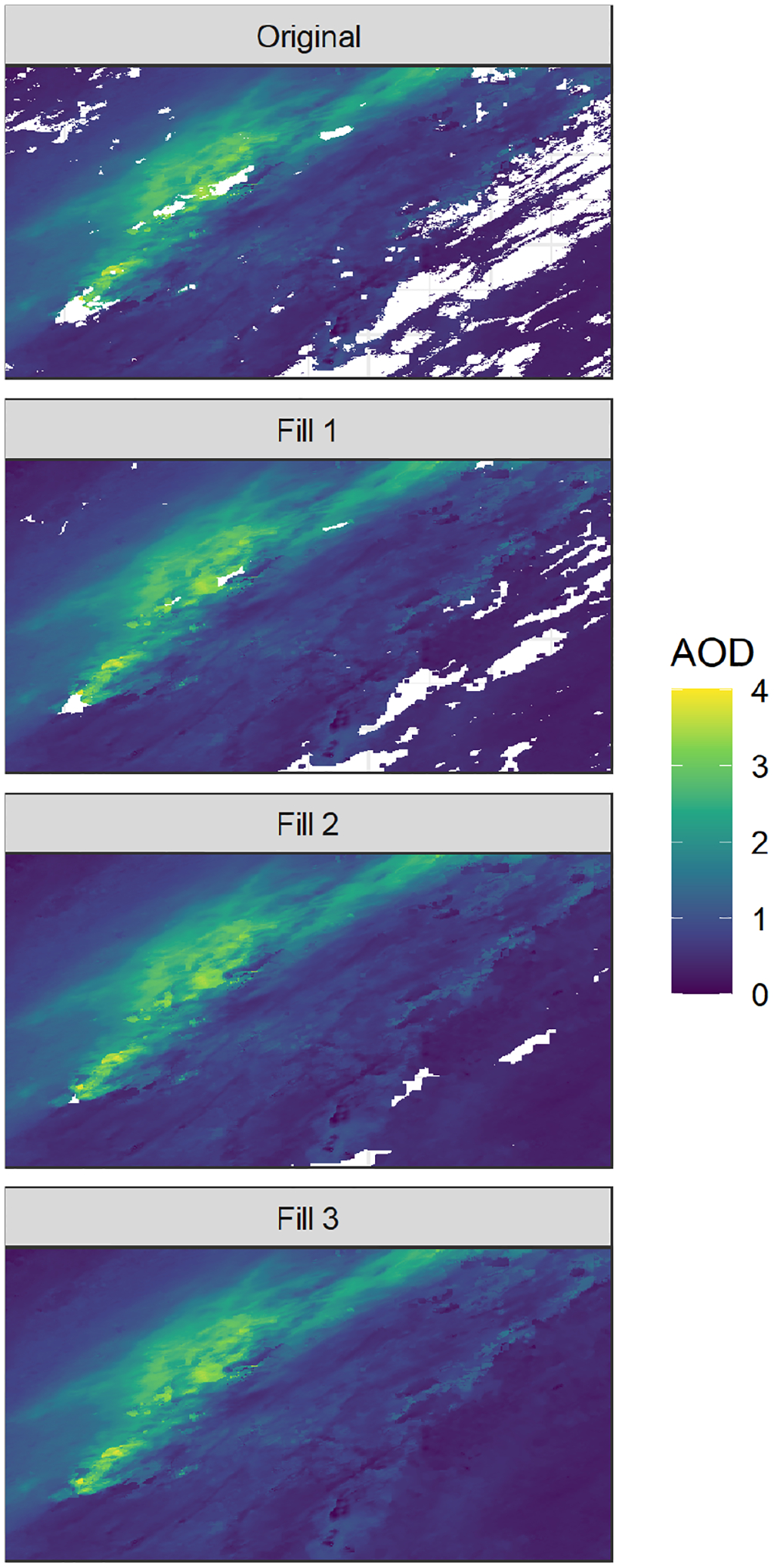
Illustration of the MAIAC AOD gap filling showing the original scene and results of three sequential focal mean imputations (denoted by Fill 1, Fill 2, and Fill 3).

The rapidfire::maiac_download function can be used to acquire the 1 km daily atmosphere product (MCD19A2) which contains AOD. Clouds prevent the retrieval of AOD, and there are sometimes clouds present even in the hot, dry conditions during California wildfires. The data fusion algorithm requires a complete data set, so a placeholder value must be used to gap-fill in locations under clouds. Previous work has used model-simulated AOD, along with meteorological variables in a data fusion approach, to gap-fill satellite-observed AOD (Zou et al., 2019). For this work, where clouds cover less of the domain, we took a simpler approach. Missing AOD values were filled using a three-stage focal average available in rapidfire::maiac_fill_gaps_complete and illustrated in Fig. 4. In the first stage, a focal mean of a 5-by-5 pixel square (5 km) is used. In the second stage, the window is increased to 9 by 9, and in the final stage it is increased to 25 by 25. Any values that are still missing after the final stage are filled with the median value for the entire scene.

#### 2.1.4 Smoke modeling

Air quality models provide near-surface estimates of PM_2.5_ on an output grid. We processed daily average PM_2.5_ concentration values acquired from the BlueSky smoke prediction system (Larkin et al., 2009) developed by the United States Department of Agriculture Forest Service (USFS) which first became operational in 2002 and has undergone significant development in recent years. The USFS runs over 30 simulations a day predicting near-surface 1 h average PM_2.5_ concentrations from wildland fire across the United States at a variety of spatial extents and resolutions using the HYSPLIT dispersion model (Stein et al., 2015). For this work we extracted BlueSky data from the California and Nevada Smoke and Air Committee (CANSAC; https://cansac.dri.edu/, last access: 10 January 2024) domain that encompasses California and Nevada for the months of July–November, years 2017–2021. In 2018 and 2019 the domain was at a 2 km resolution, and for 2019–2021 the domain was at a 1.33 km resolution. On some days, the model did not run successfully. For those days, data were backfilled by using the second or third day of a previous day’s 72 h model run. We chose this air quality data set because it is available operationally, is of a high spatial resolution, and is focused specifically on modeling smoke aerosols from wildland fires; however, other air quality modeling could be substituted.

Smoke prediction systems need to make many more assumptions than retrospective analyses. These assumptions, such as vegetation type and fuel loading, fire size and behavior, persistence of fire activity into the future, and using a meteorological forecast, all have considerable implications for the quantity of emissions released from fires and how those emissions transport and undergo chemical reactions in the atmosphere (Kennedy et al., 2020; Larkin et al., 2012; O’Neill et al., 2022). These assumptions and associated uncertainties can result in orders of magnitude spread in the estimated downwind PM_2.5_ concentrations (Li et al., 2020). Despite these issues, these systems are useful in providing information about potential smoke impacts (Lahm and Larkin, 2020), and the data are more available and can provide the underlying consistent data sets necessary to represent near-surface PM_2.5_ concentrations for successful applications of machine learning and health impact analyses. Further retrospective studies are not routinely available for long-term time periods (5–10 years or more), and maturing air quality forecasting systems, when coupled with machine learning approaches such as those provided here, can provide the consistent high-quality data sets needed for health impact analyses.

#### 2.1.5 Meteorology

Meteorological conditions can help explain the relationships between our inputs and observed PM_2.5_. For example, the PurpleAir sensor is sensitive to relative humidity. AOD is sensitive to humidity and planetary boundary layer height. Following Zou et al. (2019), we included several meteorological variables in our model, including daily average temperature, winds, humidity, boundary layer height, and daily rainfall. These variables were acquired from the North American Regional Reanalysis (NARR) data set (Mesinger et al., 2006).

### 2.2 Data fusion

We developed event-specific models using random forest (RF) regression. RF is a technique that uses a large number of randomly generated regression trees (Breiman, 2001). Each tree is constructed using a random subset of the training data, and each node uses a random subset of the potential predictive variables. New values are estimated as the mean prediction of the individual trees. For each RF run, 500 trees were grown. A single tuning parameter, the number of variables selected at each node (mtry), was varied between 2 and 5. The model was trained using 10-fold cross-validation, withholding 30 % of the monitoring data for tuning. Internally, rapidfire::develop_model uses the random-Forest R package (Liaw and Wiener, 2002).

For the final model, 10 predictor variables were used (Table 3). PM_2.5_ from the monitors was used as both a predictor and a target variable. Given a list of locations and dates, the final result from rapidfire::predict_locs is a table with the 10 input variables plus the resulting modeled PM_2.5_ for each location and date.

## 3 Results and discussion

### 3.1 Model evaluation and comparison with measurements

To demonstrate the performance of the rapidfire system, we developed models for five large wildfire smoke events from 2017–2021 in northern California (Table 1). Six quantitative analysis metrics are used to evaluate model performance (Table 4). The model was assessed in two ways.

First, a 10-fold cross-validation was performed on the permanent and temporary monitors. For each fold, 10 % of the monitoring data were withheld prior to interpolation. For this analysis, we also developed models with three simpler methods: (1) ordinary kriging (OK) interpolation of AirNow monitors, (2) OK interpolation of PurpleAir sensors, and (3) multiple linear regression (MLR) using the same inputs as those used for the rapidfire modeling.

Second, rapidfire predictions using the full data set were compared against 24 h filter-based measurements from the Interagency Monitoring of PROtected Visual Environments (IMPROVE) network and Chemical Speciation Network (CSN).

**Figure 5.**
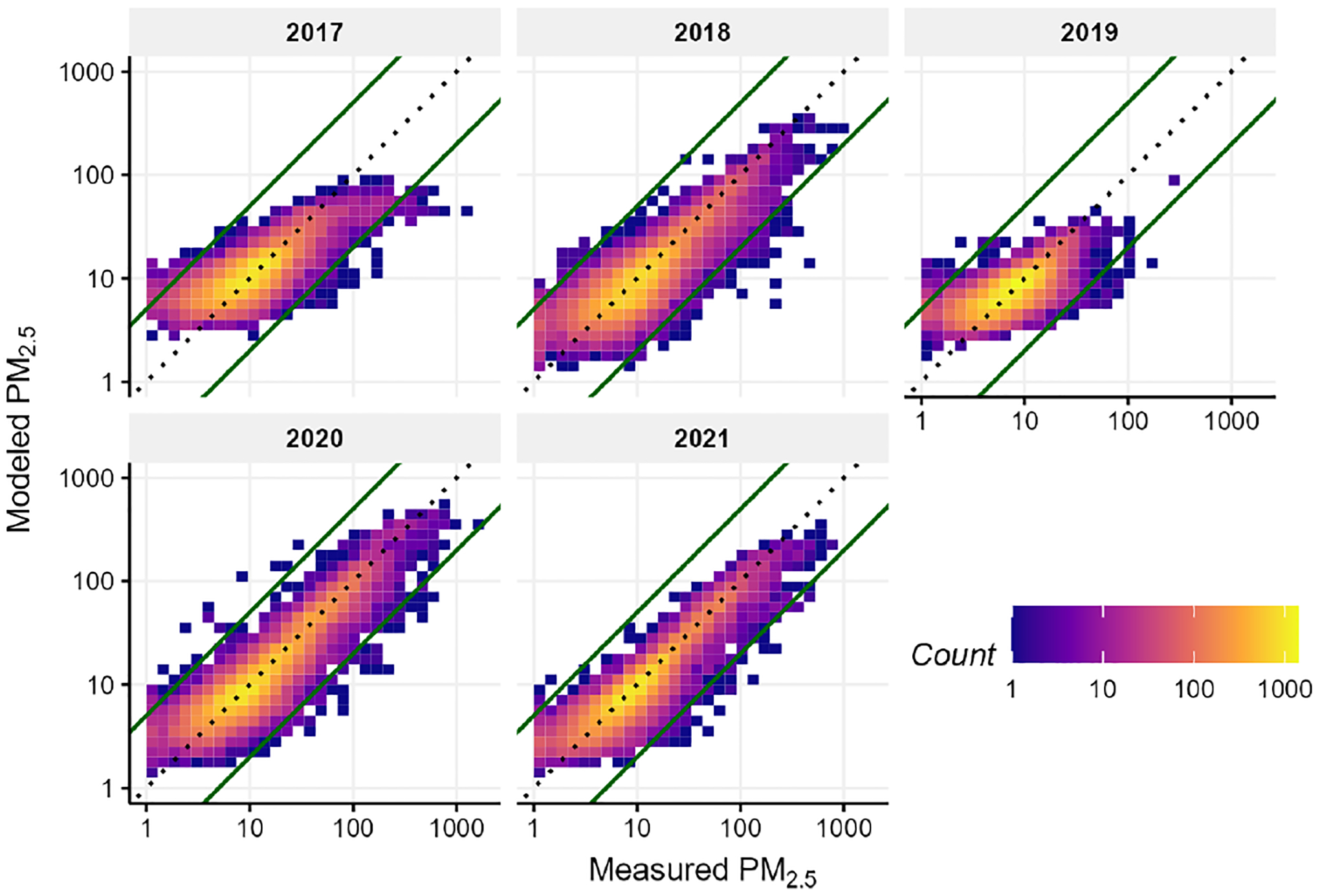
Cross-validation results by year against measured PM_2.5_ from AirNow monitors (values are given in units of μg m−3).

The cross-validation results for rapidfire are shown in Fig. 5. The vast majority of results are along the 1 : 1 line. There is a large dynamic range, with concentrations ranging from less than 1 to over 1000 μg m^−3^. The model overestimates at the lowest concentrations and sometimes underestimates the highest concentrations, especially in 2017. The relative paucity of low-cost sensors in 2017 may have contributed to poorer performance in that year.

Model performance statistics for the cross-validation using the four methods are shown in Table 5. For these wildfire events, rapidfire provides good correlation with low error and bias, offering improvement over classical MLR or interpolation of the ground monitors alone. The high density of monitors in this region helps the interpolation approaches perform well; all of the methods are available within the rapidfire package. These results are similar to results from recent data fusion studies. Cleland et al. (2020) applied bias correction and data fusion methods to estimate PM_2.5_ impacts during the 2017 wine country wildfires with a resulting correlation of 0.71. They found that temporary monitors in the more rural areas were critical in improving results. Similarly, Zou et al. (2019) applied several machine learning approaches, including random forest, to improve PM_2.5_ estimates across the Pacific Northwest (PNW) during August–September 2017, with correlations ranging from 0.45 to 0.59. Note that the PNW region is much more sparsely populated with monitors than California.

Complete rapidfire results were also compared with available observations from the IMPROVE network and CSN. Both IMPROVE and CSN collect 24 h integrated filter-based measurements of speciated particulate matter every third day (Solomon et al., 2014). IMPROVE PM_2.5_ mass is determined gravimetrically. CSN no longer performs gravimetric mass analysis, but PM_2.5_ is estimated by reconstructing total mass from the major components of PM_2.5_: ammonium sulfate, ammonium nitrate, soil, organic matter, elemental carbon, and sea salt.

**Figure 6.**
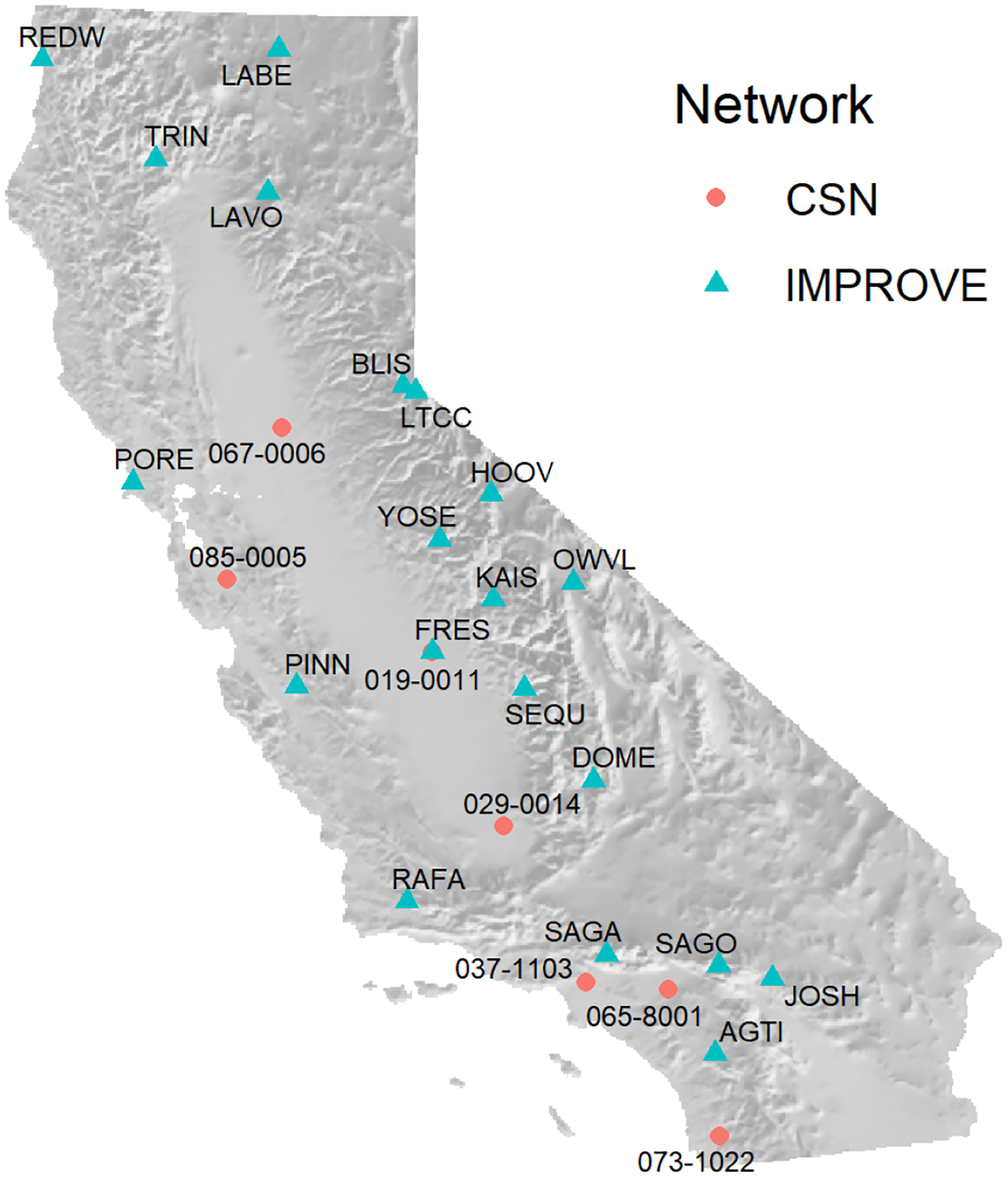
Map of CSN and IMPROVE monitoring stations used to validate model results.

**Figure 7.**
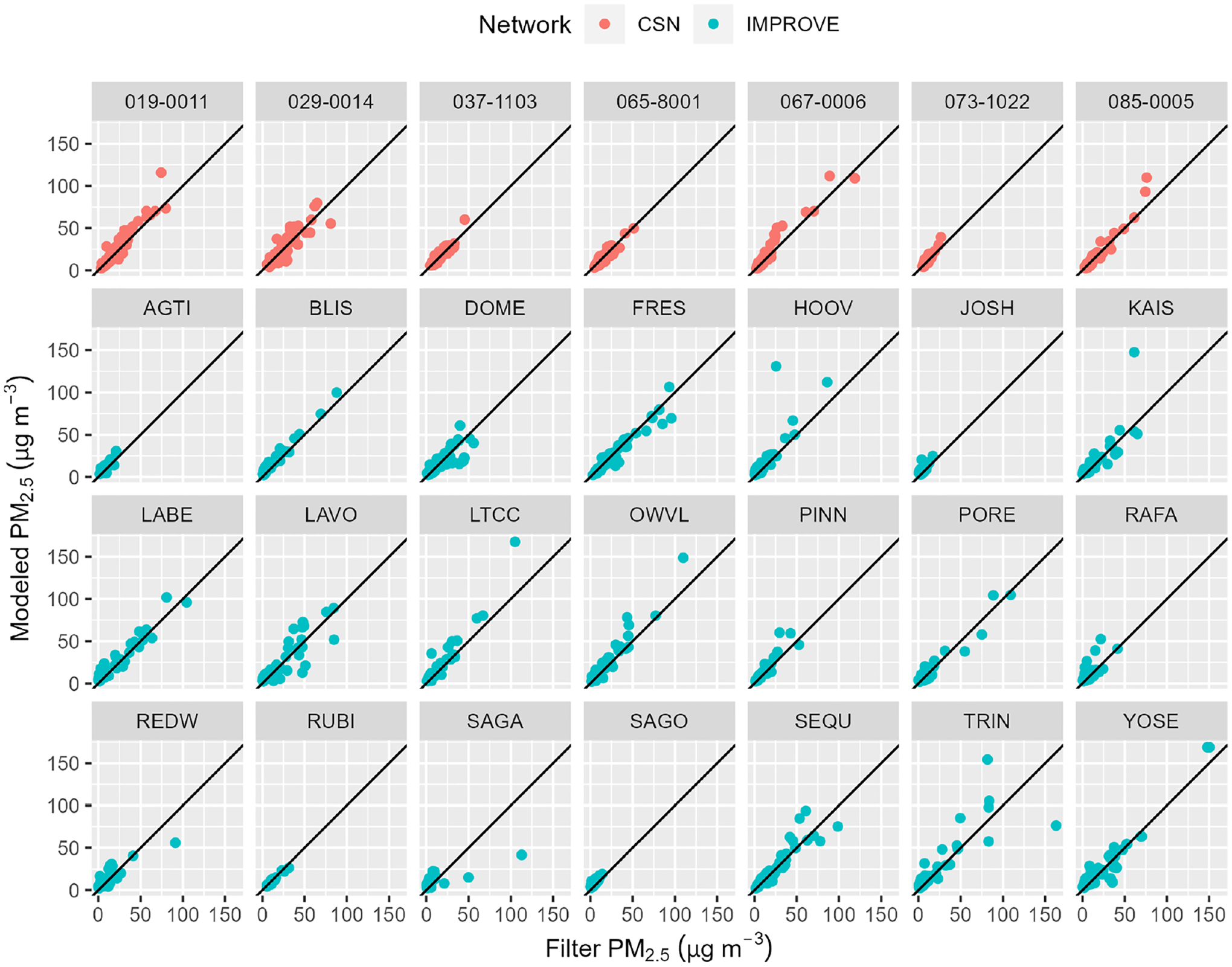
Model comparison against measured PM_2.5_ at IMPROVE and CSN monitors.

Figure 6 shows the CSN and IMPROVE monitor locations along with the identifiers used in this study. The rapidfire modeling shows excellent agreement with individual CSN and IMPROVE monitors as shown in Fig. 7 and Table 6. This is somewhat surprising, as they represent a challenging test of the method. The 24 h filter data are 100 % independent of the model inputs and, for IMPROVE especially, are located far from other monitors in remote locations with complex terrain. However, the lower dynamic range of the data helps to explain the lower RMSE compared to the cross-validation analysis above. Because the IMPROVE sampler clogs in very heavy smoke situations, the highest concentrations in this data set are less than 200 μg m^−3^. The network is also relatively sparse, and sampling is only every third day.

### 3.2 Characterizing rapidfire results across California

**Figure 8.**
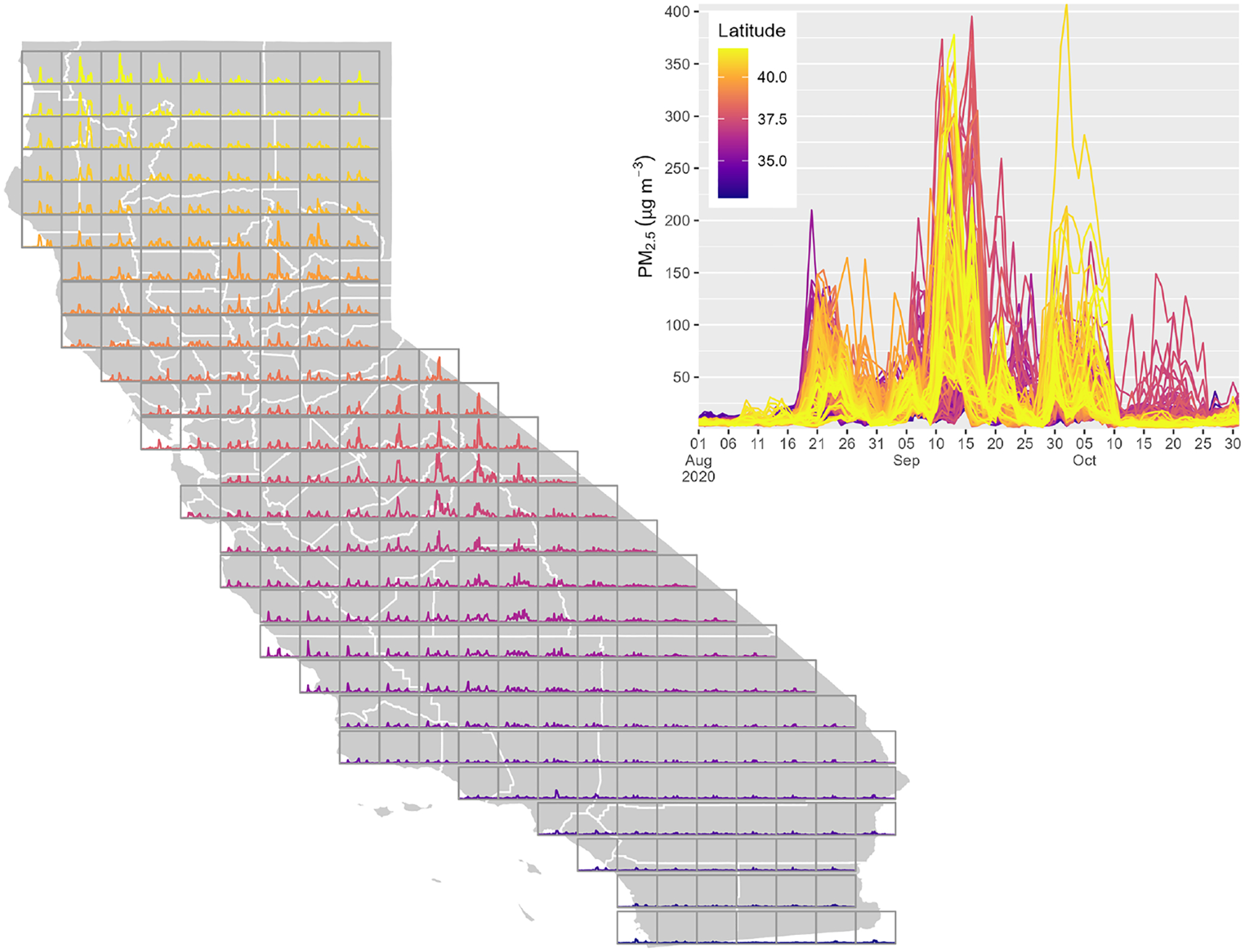
rapidfire PM_2.5_ estimates for August–October 2020. Each box on the map shows the time series for a point at the centroid of the box, and the larger plot shows all of those time series overlaid.

**Figure 9.**
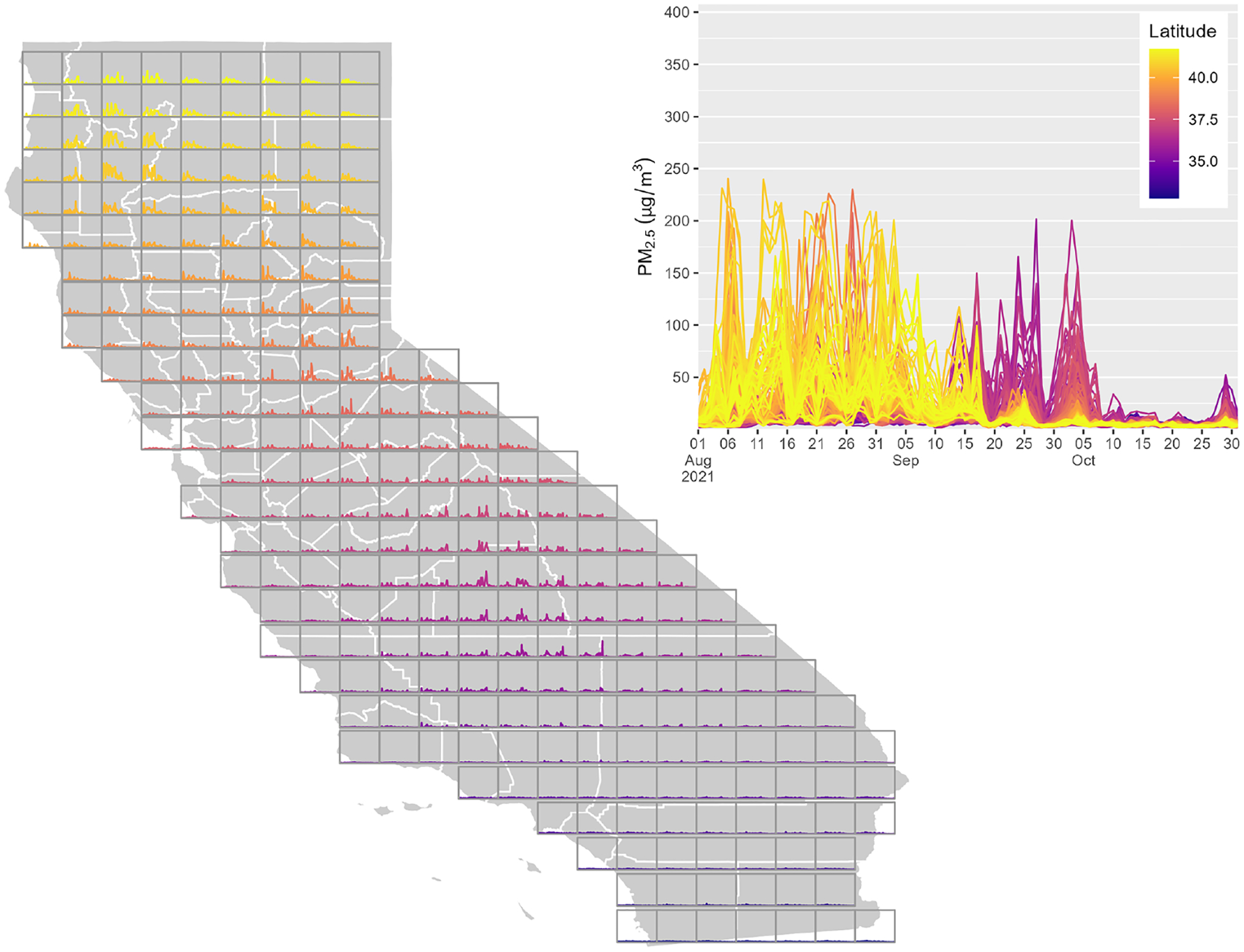
rapidfire PM_2.5_ estimates for August–October 2021. Each box on the map shows the time series for a point at the centroid of the box, and the larger plot shows all of those time series overlaid.

The results are plotted across California for two wildfire seasons: August–October 2020 (Fig. 8) and August–October 2021 (Fig. 9). In each case, daily average PM_2.5_ reaches values greater than 200 μg m^−3^, with very strong spatial and temporal variability. The 2020 case shows three widespread peaks in August, September, and October. In the 2021 case, concentrations were highest in northern locations in August, while values were higher further south in September and early October. These two cases highlight the complexity of these smoke events, which are controlled by multiple wildfires burning in and around the state simultaneously.

### 3.3 Excess mortality

As a demonstration of the utility of the rapidfire system, we adapted the methods of Johnston et al. (2012) to estimate statewide mortality attributable to excess PM_2.5_ during the wildfire seasons of 2017–2021. Excess mortality was estimated daily at the census tract level.

Mortality attributable to PM_2.5_ exposure

$$=\sum_{d=1}^{n}P\times M\times \left({\text{PM}}_{2.5,\text{d}}-{\text{PM}}_{2.5,\text{b}}\right)\times {\text{RR}}_{\text{SI}},$$

where PM_2.5,d_ is daily average PM_2.5_ concentration predicted by rapidfire at census tract centroids, with minimum and maximum values of 15 and 200 μg m^−3^. Much of California has a relatively high baseline average PM_2.5_ concentration during non-fire conditions. We developed a conservative non-fire baseline PM_2.5,b_ concentration value by taking three lower-fire-activity years (2016, 2019, and 2022) and calculating the 90th percentile of daily PM_2.5_ by month and county based on AirNow monitors. Predictions were capped at 200 μg m^−3^, as the PM_2.5_ dose–response curve flattens at higher exposures (Pope et al., 2011). *M* is the county-level daily average mortality rate, which was acquired from the Centers for Disease Control and Prevention’s WONDER database (CDC, 2023), for the year 2016 (a recent low-fire year). *P* is the census tract population from the 2020 Census (Census, 2021). RR_SI_ is the relative risk function for multiple-cause mortality due to short-term PM_2.5_ exposure. The value of RR_SI_ was 0.11 % per 1 μg m^−3^ increase in PM_2.5_ concentration (Johnston et al., 2012).

**Figure 10.**
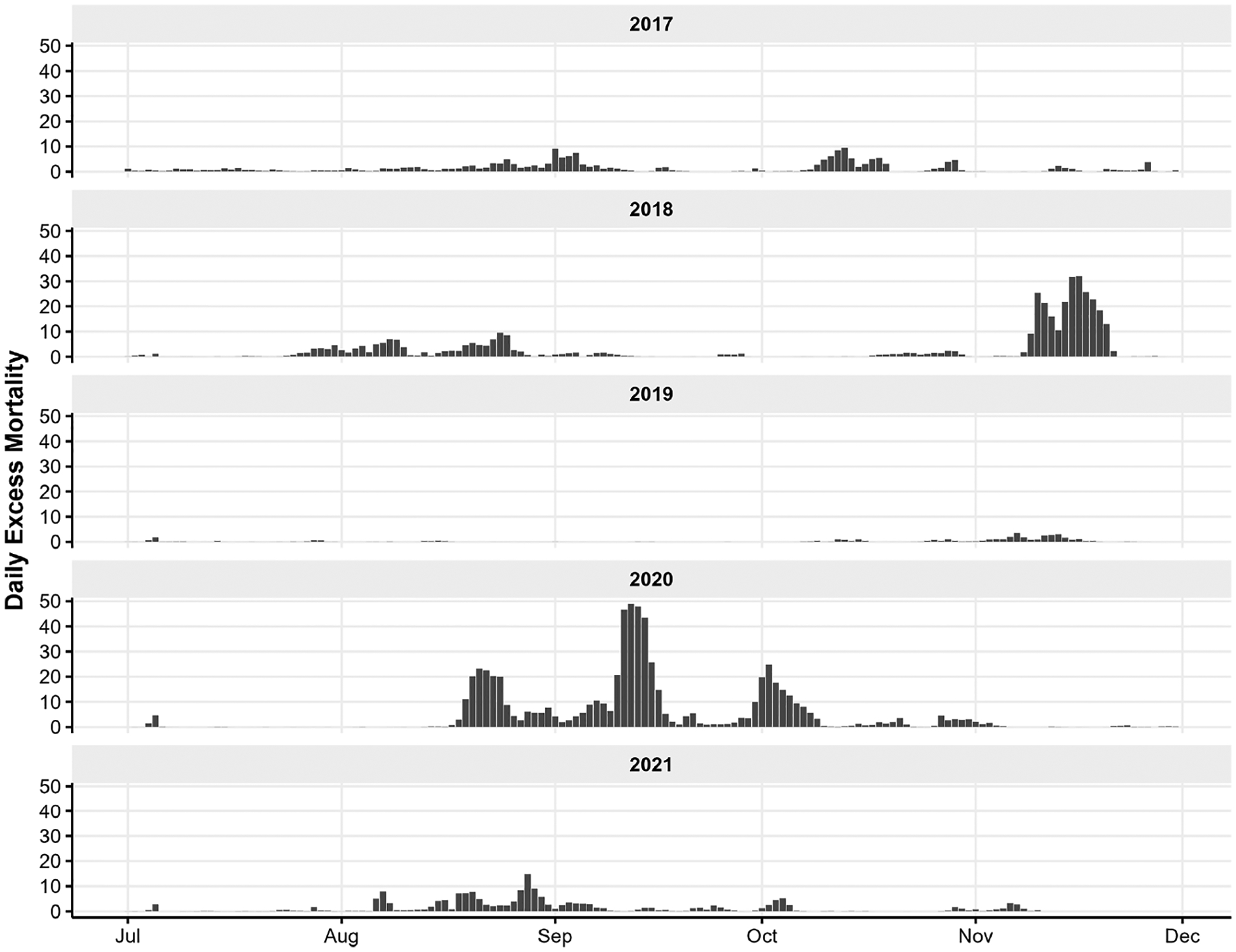
California-wide estimated daily excess mortality from PM_2.5_ concentrations above estimated baseline for the period July–November 2017–2021.

**Figure 11.**
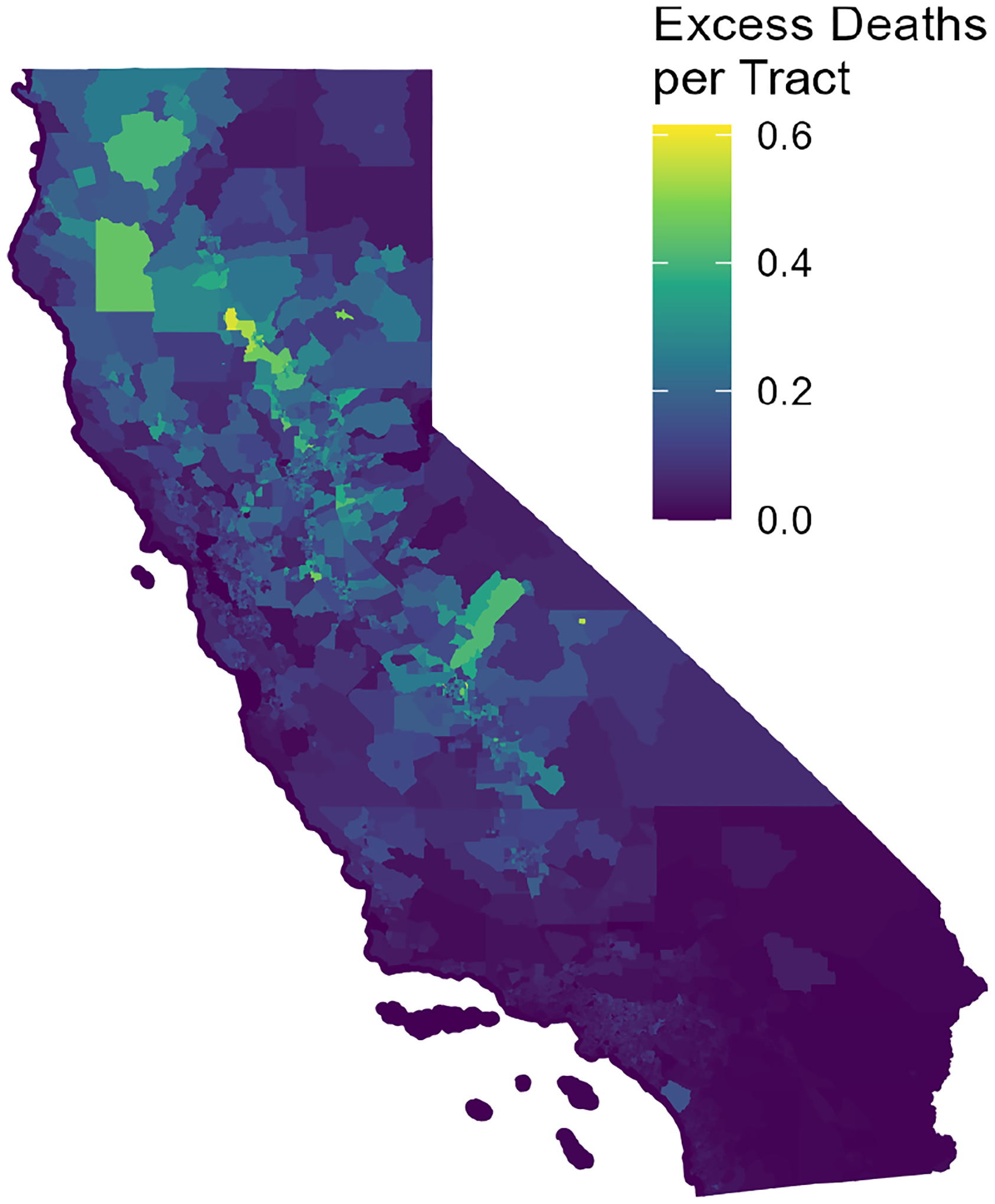
July–November 2020 excess mortality by census tract from PM_2.5_ concentrations above estimated baseline.

Figure 10 shows the California-wide daily excess mortality calculated from the increment of PM_2.5_ concentrations above PM_2.5,b_. The most significant impacts are seen in 2018 and 2020. In November 2018, the Camp wildfire produced massive PM_2.5_ emissions that were transported throughout the Sacramento and San Joaquin valleys and persisted under stagnant weather conditions. The nearly 2-week period of high concentrations across a broad region of relatively high population density led to an estimated 266 excess deaths. The historic 2020 fire season was even more dramatic. Beginning in August, smoke from fires burning around the state contributed to an estimated 615 excess deaths across a 3-month period. Incorporating the error in the rapidfire predictions, the range of excess deaths is 209–339 in the November 2018 period and 457–1072 in the 2020 3-month period. The spatial distribution of excess mortality for 2020 is shown in Fig. 11. Impacts are shown by census tract. Though census tracts vary greatly in size, they have similar populations, with a minimum of 1200 and maximum of 8000. Elevated excess mortality was widespread in the northern half of the state, especially away from the coast.

## 4 Discussion

### 4.1 Model input importance

**Figure 12.**
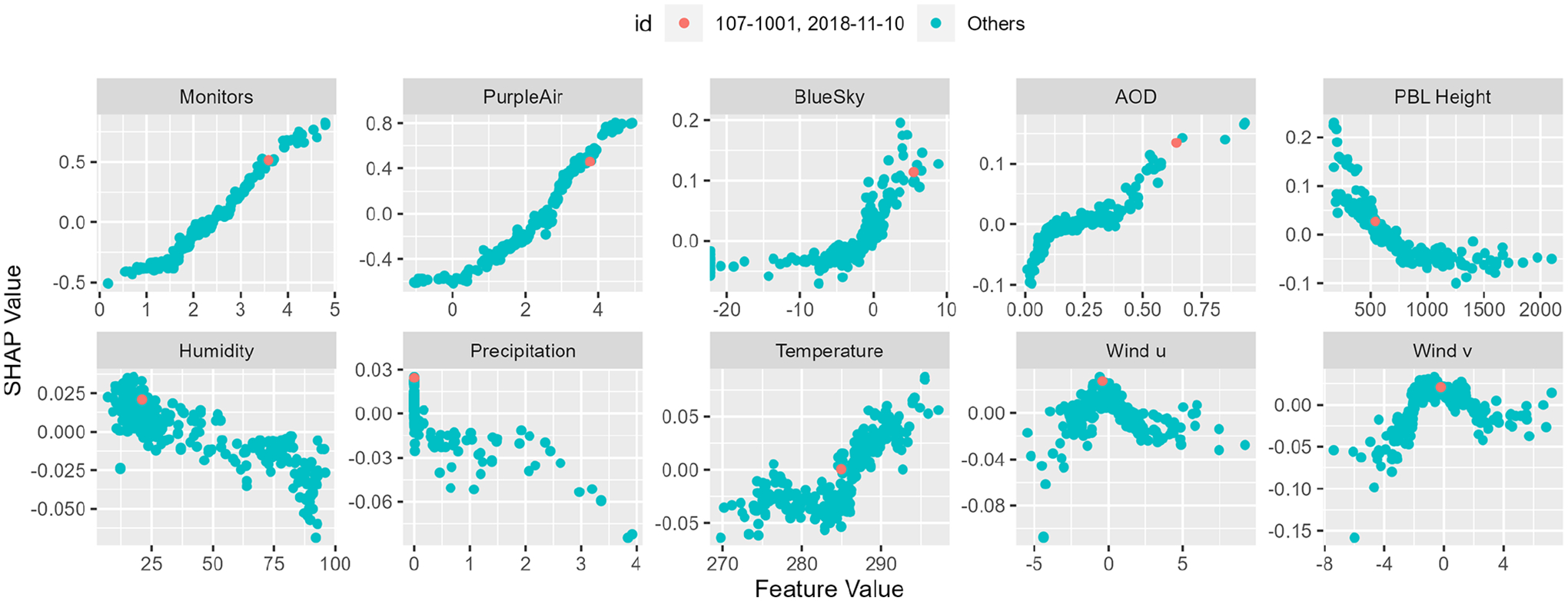
SHAP dependence plot at CSN and IMPROVE sites for 1–10 November 2018. Units for feature values depend on the variable and are listed in Table 3. BlueSky data were log-transformed in this plot for clarity.

Although the random forest model uses all of the provided predictor variables, the most explanatory variables are selected more often at each node. The relative importance of each variable can be visualized by calculating SHapley Additive exPlanations (SHAP) (Lundberg and Lee, 2017). SHAP quantifies the contribution of each predictor variable to the final model prediction. Figure 12 shows input values plotted versus SHAP for 1–10 November 2018. A single prediction, for CSN site 107–1001 on 10 November 2018, is highlighted. The SHAP values show the contributions to the final predicted concentration value from each of the model inputs. The individual component features of the model behave as expected from atmospheric dynamics. In the highlighted case, PM_2.5_ was high in the permanent and temporary monitors (Monitors), the sensor network (PurpleAir), and the smoke model (BlueSky). AOD was also elevated. By contrast, the planetary boundary layer (PBL height) was low, as were wind speeds, humidity, and precipitation. Air temperature was moderate. The magnitude of the SHAP values in Fig. 12 quantifies the relative importance of the different inputs. The ground-based networks, both official monitoring and low-cost sensors, are the most important variables in the model, followed by the BlueSky smoke model, planetary boundary height, and AOD. The remaining meteorological variables have a small but coherent impact.

### 4.2 Application for health studies

The rapidfire modeling has been applied, and is being applied, in several epidemiological studies. The ability to produce wildfire-associated PM_2.5_ measures in a timely manner (about 1 month post-event) allows time-critical planning and implementation of epidemiological studies. For example, when each of the recent large wildfires produced smoke plumes that covered urban areas of northern California, the rapidfire modeling was used to determine the time periods and geographical areas where populations were most impacted by wildfire smoke. This information was used in two local studies, the Bio-Specimen Assessment of Fire Effects (B-SAFE) wildfire pregnancy cohort study and the WHAT-Now CA wildfire cohort study, to recruit participants from highly affected areas to collect information and biological specimens to analyze later for wildfire-associated compounds and biologic responses as indicators of potential for downstream health impacts. Both studies also related the wildfire-associated PM_2.5_ from rapidfire modeling to reported symptoms and health outcomes of the cohort participants. In B-SAFE, the timing and concentrations of PM_2.5_ are being linked to birth outcomes of the children gestationally exposed to wildfires for the initial study and in follow-up studies on respiratory, developmental, and other child conditions. Specimens collected in B-SAFE for those with higher versus lower modeled wildfire-associated PM_2.5_ are also being compared across various measures (e.g., metals, contaminants, cytokines) to better understand differences by degree of exposure. In WHAT-Now CA, PM_2.5_ is being examined in association with respiratory outcomes. Both studies are planning to follow these exposed cohorts forward to examine later health outcomes.

Other local studies, including existing cohorts not focused on wildfire exposure, like the Markers of Autism Risk in Babies – Learning Early Signs (MARBLES) pregnancy cohort study of younger siblings of children with autism (Hertz-Picciotto et al., 2018), also used the rapidfire modeling in order to identify mothers and infants exposed to wildfire smoke during pregnancy and examine specimens being collected as part of the protocol for differences. Further, outcomes of these children, who are at higher risk of autism and other neurodevelopmental conditions, will be compared across those exposed and unexposed to wildfire.

rapidfire modeling will be used to determine the time periods and geographical areas where populations were and will be most impacted by future wildfire smoke events for other statewide air pollution studies, including one funded by EPA (EPA STAR 84048401) that will link air pollution measures, including wildfire-specific air pollution, to birth outcomes and neurodevelopmental disorders and work with the most affected communities to distribute education, materials, and tools for mitigating exposures.

### 4.3 Advantages over existing methods

There are many methods to produce spatially resolved estimates of PM_2.5_ for use in exposure studies. The advantages of rapidfire include reliance on only off-the-shelf inputs with low latency, inclusion of data sets that provide improvements for wildland fire smoke, and an extensible framework with an open code base. If a new smoke event occurred, all inputs would be accessible and PM_2.5_ modeling could be completed within 1 month. At present, only the NARR meteorological data are not available in near real time. In future work, these could be replaced by a daily operational model and the rapidfire predictions could be produced 1 d after an event. The addition of a low-cost sensor network has also significantly improved resulting predictions. The rapidfire algorithm and code base have been designed to be modular so that new inputs can be included as they become available. For example, the MAIAC AOD may become unavailable as the MODIS instrument reaches end of life. A new function could be added to deal with AOD from another data source.

### 4.4 Limitations and future directions

The rapidfire modeling approach has some limitations. The model requires high-quality training data to produce a high-quality result. In areas without accurate PM_2.5_ measurements at point locations within the modeling domain, there is no way to create a reliable regression, though this is true for all statistical air quality models. In this study, the monitors from the AirNow network served that purpose. However, AirNow is only present in the United States, and the current rapidfire functions require data sets that are not all globally available. These data sets could be replaced by others to cover a specific region, and new handling functions could be added to rapidfire to support those data sets as needed.

The rapidfire methods are designed with wildfire smoke events in mind. They are best suited for regional-scale modeling at spatial resolutions of 1 km or larger. This is appropriate for smoke events, which are driven by a regional source that impacts a broad swath. rapidfire would be less suitable for modeling exposure to PM_2.5_ from emission sources at very fine spatial scales, such as near-road emissions. Also, rapidfire is currently limited to estimates of total PM_2.5_ only. Estimates of PM_2.5_ composition, or specific wildfire contribution, are not supported with the currently available inputs, though this is an area of future work.

The random forest regression method has historically been seen as a black box, with potential for good prediction but limited ability to provide insight into the drivers of the model prediction and the underlying physical phenomena. However, the advent of new metrics for explaining machine learning models, such as SHAP, makes these models more useful and transparent.

Several improvements could be made to enhance the algorithm and potentially improve performance. The recently released collection 6.1 of MAIAC AOD provides better spatial coverage and more accurate results in conditions of heavy smoke compared to collection 6.0 (Ye et al., 2022). The relatively simplistic gap-filling approach applied to AOD should be reviewed, especially for use in cloudier conditions. Additional transport models with modern fire emissions processing and broad coverage, such as HRRR-Smoke (https://rapidrefresh.noaa.gov/hrrr/HRRRsmoke/, last access: 10 January 2024), could be tested. Other machine learning algorithms such as eXteme Gradient Boosting (XGBoost) should be explored.

## 5 Conclusions

The rapidfire R package was developed to model relatively accurate particulate information derived from inputs retrieved easily. It incorporates off-the-shelf data sets that are produced operationally and with low latency (< 1 month) within a machine learning framework. rapidfire takes advantage of the recent burgeoning of low-cost sensors around the world, in addition to traditional air pollution data sources such as ground-based monitoring networks and satellitederived aerosol products. The rapidfire code is available for use and contribution at https://github.com/raffscallion/rapidfire (last access: 10 January 2024). We demonstrated rapidfire modeling for five recent wildfire seasons in California and validated results against fully independent filter-based measurements of PM_2.5_. rapidfire showed excellent performance, predicting PM_2.5_ under heavy smoke with high accuracy, even at remote and elevated sites. An example calculation of conservative excess mortality from high PM_2.5_ exposure in California showed large impacts, including an estimated 615 excess deaths in California over a 3-month period of intense wildfire smoke in 2020. rapidfire PM_2.5_ estimates are currently being used in several health effect studies in California. In the future, we hope to expand the methods to include data sets that are of even lower latency. At present, the input that becomes available the most slowly is the NARR meteorology, which is available at the end of each month. There are several candidate meteorological data sources that are available daily, which would allow for next-day estimates of PM_2.5_. These low-latency estimates would be useful for rapid deployment, recruitment, and sample collection in epidemiologic studies.

## Figures and Tables

**Table 1.** Modeled time periods and major California wildfires. Annual area burned in California is from the United States National Interagency Fire Center (NIFC; https://www.nifc.gov/fire-information/statistics, last access: 10 January 2024).

Year	Time period	Major California fires	Annual California area burned (ha)
2017	Oct	Atlas, Nuns, Pocket, Redwood Valley, Tubbs	513 000
2018	15 Jul–15 Sep; Nov	Carr, Mendocino, Ferguson, Camp, Woolsey	738 000
2019	15 Oct–15 Nov	Kincade	105 000
2020	Aug–Oct	August, Apple, Creek, Dolan, Dome, LNU Lightning, North, SCU Lightning, Bobcat	1 657 000
2021	Aug–Oct	Antelope, Caldor, Dixie, Monument, River	905 000

**Table 2.** Data sources used in rapidfire and the rapidfire function to access them or the location where sample data are available.

Data source	rapidfire function or location where available	Spatial resolution
AirNow permanent PM_2.5_ monitoring data	rapidfire::get_airnow_daterange	Point locations
IWFAQRP temporary PM_2.5_ monitoring data	rapidfire::get_airsis_daterange	Point locations
PurpleAir air sensor data	rapidfire::openaq_get_averages	Point locations
MAIAC aerosol optical depth	rapidfire::maiac_download	1 km
Example smoke modeling data	https://doi.org/10.5281/zenodo.7942846 (Raffuse and O’Neill, 2023)	4 km
North American Regional Analysis (NARR) meteorology	rapidfire::get_narr	32 km

**Table 3.** Predictor variables used in the rapidfire RF model.

Variable	Name	Description	Units
PM25_log_ANK	Monitors	Log-transformed, interpolated PM_2.5_ from permanent and temporary monitors	μg m^−3^
PM25_log_PAK	PurpleAir	Log-transformed, interpolated PM_2.5_ estimates from PurpleAir sensors	μg m^−3^
PM25_bluesky	BlueSky	Daily average ground-level PM_2.5_ predictions from BlueSky smoke model	μg m^−3^
MAIAC_AOD	AOD	Gap-filled daily AOD from MAIAC	Unitless
air.2m	Temperature	Daily average ambient temperature at 2 m above ground level (a.g.l.) from NARR	K
uwnd.10m	Wind *u*	Daily average *u* component of wind at 10 m a.g.l. from NARR	m s^−1^
vwnd.10m	Wind *v*	Daily average *v* component of wind at 10 m a.g.l. from NARR	m s^−1^
rhum.2m	Humidity	Daily average relative humidity at 2 m a.g.l. from NARR	%
apcp	Precipitation	Daily total precipitation amount from NARR	cm
hpbl	PBL height	Daily average height of the planetary boundary layer from NARR	m

**Table 4.** Definitions of quantitative analysis metrics.

Metric	Equation
*r* ^2^	$\frac{\sum_{i}{\left({\hat{Y}}_{i}-\bar{Y}\right)}^{2}}{\sum_{i}{\left(Y_{i}-\bar{Y}\right)}^{2}}$
Root-mean-square error (RMSE)	$\sqrt{1-r^{2}}{\text{SD}}_{Y}$
Median bias	$\text{med}\left({\hat{Y}}_{i}-Y_{i}\right)$
Normalized bias (%)	$100\cdot \text{med}\left(\frac{{\hat{Y}}_{i}-Y_{i}}{Y_{i}}\right)$
Median error	$\text{med}\left(\frac{{\hat{Y}}_{i}-Y_{i}}{Y_{i}}\right)$
Normalized error (%)	$100\cdot \text{med}\left(\text{abs}\left(\frac{{\hat{Y}}_{i}-Y_{i}}{Y_{i}}\right)\right)$

**Table 5.** Performance metrics for four modeling methods.

Model	*R* ^2^	RMSE	Median bias	Normalized bias	Median error	Normalized error
rapidfire	0.87	16.1	−0.08	−0.76	−0.008	18.0
MLR	0.84	17.6	0.01	0.11	0.001	22.6
AirNow OK	0.80	19.5	−0.03	−0.26	−0.002	23.4
PurpleAir OK	0.80	19.4	1.69	15.2	0.152	41.1

**Table 6.** Performance metrics for rapidfire at AirNow, IMPROVE, and CSN sites.

Network	*R* ^2^	RMSE	Median bias	Normalized bias	Median error	Normalized error
CSN	0.87	5.18	0.42	3.93	1.96	15.3
IMPROVE	0.81	8.47	2.48	46.5	3.19	49.6

## Data Availability

The current version of rapidfire is available on the project website: https://github.com/raffscallion/rapidfire (last access: 10 January 2024) under the license GPLv3. The exact version of the model used to produce the results used in this paper (v0.1.3) is archived on Zenodo (https://doi.org/10.5281/zenodo.7888562; Raffuse, 2023), as are input data and scripts to run the model and produce the plots for all the simulations presented in this paper (https://doi.org/10.5281/zenodo.7942846; Raffuse and O’Neill, 2023).
